# Enzymatic Hydrolysis and Fermentation of Banana Pseudostem Hydrolysate to Produce Bioethanol

**DOI:** 10.1155/2021/5543104

**Published:** 2021-07-13

**Authors:** Lesetja Moraba Legodi, Daniel Coenrad LaGrange, Elbert Lukas Jansen van Rensburg, Ignatious Ncube

**Affiliations:** University of Limpopo, Department of Biochemistry Microbiology and Biotechnology, Private Bag X 1106, Sovenga 0727, South Africa

## Abstract

Banana pseudostem (BPS) is an agricultural waste with a high holocellulose content, which, upon hydrolysis, releases fermentable sugars that can be used for bioethanol production. Different pretreatment methods, namely, 3% (w/v) NaOH, 5% (v/v) H_2_SO_4_, and liquid hot water, applied on the BPS resulted in the availability of 52%, 48%, and 25% cellulose after treatment, respectively. Saccharification of the pretreated BPS with 10 FPU/g dry solids (29.3 mg protein/g d.s) crude enzyme from *Trichoderma harzianum* LMLBP07 13-5 at 50°C and a substrate loading of 10 to 15% released 3.8 to 21.8 g/L and from *T*. *longibrachiatum* LMLSAUL 14-1 released 5.4 to 43.5 g/L glucose to the biomass. Ethanol was produced through separate hydrolysis and fermentation (SHF) of alkaline pretreated BPS hydrolysate using *Saccharomyces cerevisiae* UL01 at 30°C and 100 rpm. Highest ethanol produced was 17.6 g/L. Banana pseudostem was shown as a potentially cheap substrate for bioethanol production.

## 1. Introduction

The production of first-generation bioethanol using grain or vegetable oil for biodiesel is faced with challenges due to competition with food production, particularly in developing countries [1]. Due to the direct impact sugar-rich feedstocks have on food prices, it has become imperative for future biofuel expansion to be based on the use of lignocellulosic biomass, a second-generation feedstock [2]. Lignocellulosic biomass comprises forestry, agroindustrial, and food waste and is renewable, available in excess, and inexpensive [3]. Agroindustrial residues are obtained after harvesting [4]. Lignocellulose mainly consists of sugar polymers (i.e., cellulose and hemicellulose) and lignin. The use of these polymers for the production of value-added products, such as biofuel, food additives, organic acids, and enzymes, can remediate environmental problems attributed to waste accumulation [3].

Cellulose polymer contains amorphous and crystalline regions and naturally exists in its crystalline form, which renders its resistance to enzymatic hydrolysis and therefore yields low concentrations of monosaccharide sugars. The crystalline form of cellulose is produced when several cellulose chains are held together by covalent bonds, hydrogen bonds, and van der Waals forces forming microfibrils. These microfibrils are bundled together to form cellulose fibres, making cellulose an ultrastructure [5]. Hemicellulose is the second most abundant polymeric carbohydrate in plant materials and renewable carbon with the potential to be converted into liquid fuels and chemicals. Hemicelluloses are heterogeneous polymers, which are easily hydrolysed by acids to their monomeric components made up of pentoses (D-xylose and D-arabinose), hexoses (D-glucose, D-mannose, and D-galactose), and sugar acids [6]. Lignin is the third plant polymer that provides the flexibility and strength required by plants. Lignin is an aromatic and hydrophobic polymer synthesised from one, two, or three different phenylpropanoids, namely, *p*-coumaryl alcohol (*p*-hydroxyphenyl propanol), sinapyl alcohol (syringyl propanol), and coniferyl alcohol (guaiacyl propanol), which are singly derived from the amino acid phenylalanine through enzymatic processes [7].

Due to the nature and structure of cellulose in plants, including herbaceous plants, the release of fermentable sugars poses challenges [8, 9]. Pretreatment step is necessary to overcome the recalcitrance of biomass and make the polymers available for subsequent hydrolysis and fermentation [10]. By definition, pretreatment is a prehydrolysis step that exposes cellulose fibres and makes it susceptible to enzymatic hydrolysis (breaking down of cellulose to fermentable sugars) before fermentation of the sugars released [11, 12]. The efficiency of pretreatment is influenced by the methods used and the nature and composition of the lignocellulosic feedstock [13]. The pretreatment of lignocellulosic biomass, which can be done through the use of chemical, physical/mechanical, biological, and physicochemical or combination of methods, enables the biomass to be porous and easily accessible for microbial growth in SSF and susceptible to enzymatic hydrolysis. The hydrolysis reaction increases the production of fermentable sugars that can be converted through biochemical activities of fermenting microorganisms into ethanol [14]. Currently, the amount of cellulases required for hydrolysis (i.e., saccharification) and the low ethanol yield are key factors affecting the overall cost of producing bioethanol [15].

Physical pretreatment involves particle size reduction by milling or grinding in order to improve enzymatic hydrolysis. Chemical pretreatment methods use acid or alkaline solutions. Acid hydrolysis is one of the most promising methods, usually performed with mineral acids, but other organic acids and sulphur dioxide may be used. It results in high recovery of hemicellulosic sugars in the pretreatment liquid and in a solid cellulose fraction with enhanced enzymatic convertibility [16]. Liquid hot water (LHW) is an environmentally friendly approach in which water in the liquid phase is used to pretreat lignocellulosic biomass. It is a relatively mild pretreatment method that does not require any catalysts and does not cause significant corrosion problems on the reactor. The solubilisation of hemicellulose is catalysed by hydronium ions resulting from water autoionization [16]. Alkaline treatment is efficient for removing lignin and increases the digestibility of cellulose. Compared to acid and LHW, mild alkaline pretreatments lead to less solubilisation of hemicelluloses and less formation of inhibitory compounds, and the pretreatments can be carried out at lower temperatures [16].

Recent developments for bioeconomy establishment have recognised biorefinery as an innovative and efficient approach to fully utilise the available biomass resources for the synergistic coproduction of power, heat, and biofuels alongside food and feed ingredients, chemicals, etc., for large-scale sustainable bioeconomy [17]. Biomass feedstock is a starting material in the development of bioeconomy, and it determines the viability of any biomass processing activities, and sustainable supply of biomass is crucial to the success of bio-based economy [17]. In this study, banana pseudostem is identified as an attractive, nonedible, cellulosic feedstock suitable for bioethanol production because of its high cellulose, moderate hemicellulose, and low lignin contents. Unlike wood-based lignocellulosic biomass, the banana plant has a short life cycle, requiring only 10–14 months to bear fruits depending on the geographic location and soil type [18]. The banana plant bears fruits once in its life cycle, and for every cycle of banana production, there are four times of waste generated, higher than the harvested fruit. Banana lignocellulosic rich waste includes fruit-bunch stems, leaves, pseudostem, and rhizome [19, 20]. After the harvesting of the banana fruit, BPS makes about 75% of the total waste generated, which is either left to rot at the local dumpsite or left to decompose in the plantation to serve as an organic soil fertiliser [20–22]. Converting BPS into reducing sugars for bioethanol production is often incomplete due to the high crystallinity of the banana cellulose fibres [13]. This study investigates different pretreatment methods on BPS, which is a source of sugars that can be used in the production of bioethanol.

## 2. Materials and Methods

### 2.1. Collection and Preparation of the Banana Pseudostem

Fresh BPS was cut, collected from Allesbeste farm located in Tzaneen (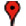 23.800943°S 30.123264°E 799 m M1 R71 Tzaneen 0850), Limpopo Province, South Africa. The BPS was washed to remove soil and other debris using tap water. Washed BPS was cut into small pieces (approximately 20–25 cm in diameter and 10 cm in height), and the outer and core bark (pith) were separated into several blocks. The blocks and piths were dried in an oven at 65–70°C (Scientific, Digital model no. 276) until a constant mass. The dried BPS blocks and piths were subjected to grinding using a milling and crushing machine (Zhuans, Electric model). The ground material was sieved through a universal test sieve with an aperture size of 500 *μ*m [19]. The sieved ground particles (<500 *μ*m) were stored at room temperature (20–25°C) in a sealed container until needed.

### 2.2. Pretreatment of the Banana Pseudostem

**Figure 1:**
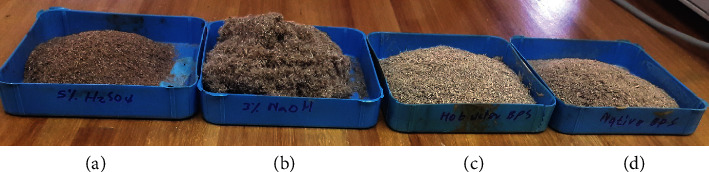
Banana pseudostem before and after pretreatment. (a) 5% H_2_SO_4_, (b) 3% NaOH, (c) hot water, and (d) untreated.

One hundred and fifty grams of ground BPS was suspended in each pretreatment solution, i.e., 3% NaOH [23], 5% H_2_SO_4_ [13, 24, 25], and liquid hot water, LHW-autoclave, at a ratio of 1 : 10 solid : liquid. The slurries were autoclaved at 121°C at 15 psi for 1 hour according to Kim [26] (HL-340 Vertical Type Steam Sterilizer, Taiwan) and cooled prior to decanting and filtration of liquid through folded gauze cloth. The subsequent step was washing the insoluble solid material with tap water until the slurry reaches pH 7.0. The filtrate fraction from different pretreatments was analysed for sugars using high-performance liquid chromatography (HPLC). Two millimetre samples were placed in a boiling water bath for 15 minutes and subsequently centrifuged at 12,470 ×g for 10 minutes (Beckman Coulter Microfuge^®^ 16 centrifuge). The solid material was dried in an oven at 65–70°C until constant mass and ground using a Waring commercial blender (model 32BL8) (Figure 1).

The ground pretreated BPS materials were stored at room temperature in a sealed container until needed. The effect of pretreatment determined by mass changes in the BPS was done by calculating the percentage of solubilised solid material using the equation described by Matsakas et al. [27]:

(1) $$\begin{array}{c} \text{component solubilisation }\left(\text{\% w}/\text{w}\right)\\ =\left(\frac{w_{\text{untreated }}\times \;x_{\text{untreated}}-w_{\text{pretreated }}\times \;x_{\text{pretreated}}}{w_{\text{untreated }}\times \;x_{\text{untreated}}}\right)\times 100\;, \end{array}$$

where *w*_untreated_ and *w*_pretreated_ are the dry masses of the untreated and recovered pretreated solids, respectively, and *x*_untreated_ and *x*_pretreated_ represent the component (cellulose, hemicellulose, and lignin) biomasses in % w/w.

### 2.3. Chemical Composition of the Banana Pseudostem

The chemical composition of the untreated, thermo-alkali, thermo-acid, and liquid hot water (or hydrothermal) pretreated BPS samples was determined. The cellulose and lignin content was analysed by a reaction with sulphuric acid according to a standard method by TAPPI-T222 om-88, and the hemicellulose content was obtained as described in TAPPI1T19m-54 standards [28].

### 2.4. Fourier-Transform Infrared Spectroscopy (FTIR) of Banana Pseudostem Fibres

Fourier-transform infrared spectroscopy of untreated, thermo-alkali, thermo-acid, and hydrothermally pretreated BPS samples was performed using a Spectrum 100 FT-IR (PerkinElmer, Waltham, MA, USA) equipped with an attenuated total reflection (ATR) accessory with a diamond/ZnSe crystal (64 scans, 4 cm^−1^ resolution, with wavenumber range 500–4000 cm^−1^) according to Motaung and Anandjiwala [28].

### 2.5. Cellulase Production and Extraction

Two fungal species, namely, *Trichoderma harzianum* LMLUL 13-5 and *Trichoderma longibrachiatum* LMLUL 14-1, obtained from the University of Limpopo culture collection, South Africa, Legodi et al. [29], were used for the production of cellulases in solid-state fermentation. Three grams of untreated BPS was moistened to 75% with a synthetic medium, as described by Peixoto [30], in 250 ml Erlenmeyer flasks. The synthetic medium consisted of 2 g/L K_2_HPO_4_, 0.5 g/L KCl, 0.01 g/L FeSO_4_·7H_2_O, 0.15 g/L MgSO_4_·7H_2_O, 7 g/L g KH_2_PO_4_, 1 g/L (NH_4_)SO_4_, and 1.2 g/L yeast extract. pH of the medium was adjusted to 6.5 using 1 M NaOH or 1 M HCl prior to sterilization at 121°C and 15 psi for 15 min (HL-340 Vertical Type Steam Sterilizer, Taiwan). A 1 mL spore suspension (1 × 10^8^ spores/ml) of either *T. longibrachiatum* LMLSAUL 14-1 or *T. harzianum* LMLBP07 13-5 was inoculated into separate 250 ml Erlenmeyer flasks (in triplicate) each containing 3 g of untreated BPS. The flasks were incubated at 30°C for 9 days without shaking (Incubator Shaker Series, New Brunswick, Excella E25R). The whole content of a flask was sampled from day 3 up to day 9.

Enzyme extraction was carried out by the modified method described by El-Shishtawy et al. [31]. Crude enzyme was extracted by adding 50 mL of 0.05 M sodium citrate buffer (pH 4.8) to the fermented contents in the flasks with intermittent shaking for 1 hour at room temperature (20–25°C). The mixture was filtered and centrifuged at 3834 ×g for 10 min (Beckman Coulter Allegra X-22R refrigerated benchtop centrifuge), and the supernatant was used for enzyme assays. Filter paper activity was used to determine total cellulase activity of the crude enzyme using the following equation:

(2) $$\begin{array}{c} \text{FPA}\left(\frac{\text{FPU}}{\text{mL}}\right)=\frac{0.37}{\left[\text{Enz}\right]}, \end{array}$$

where FPU is the filter paperase activity unit and [Enz] is the concentration of the enzyme that releases 2.0 mg of glucose from the 50 mg filter paper in 60 minutes under the conditions of the assay [32]. For cellulase activity in SSF, FPU/mL was converted into FPU/g d.s (d.s refers to dry substrate) using the following equation [33]:

(3) $$\begin{array}{c} \text{FPA }\left(\frac{\text{FPU}}{\text{g d}.\text{s}}\right)=\frac{\left(\text{FPU}/\text{mL}\right)\times \text{total volume of the fungal extract }\left(\text{mL}\right)}{\text{dry weight of the substrate used in SSF }\left(\text{g}\right)}. \end{array}$$

### 2.6. Enzymatic Hydrolysis of the Pretreated Banana Pseudostem

Crude cellulases from *T*. *longibrachiatum* LMLSAUL 14-1 and *T*. *harzianum* LMLBP07 13-5 cultures were used for enzymatic hydrolysis. The hydrolysis of alkaline (3% NaOH), acid (5% H_2_SO_4_), and LHW pretreated BPS at 10 g, 12.5 g, and 15 g (i.e., 10, 12.5, and 15%) solid loadings in 100 mL 0.05 M sodium citrate (pH 5.0) was carried out in triplicate at 50°C and 150 rpm for 76 hours. The hydrolysis reaction mixture contained crude enzyme (10 FPU/g of the substrate equivalent to total protein concentration of 29.3 mg/g substrate, Yang et al. [34] and Gregg and Saddler [35]) and 0.005% sodium azide. Sampling was carried out according to Low et al. [36]. Sugar analysis was performed according to the procedure followed under the pretreatment section.

### 2.7. Sugar Analysis in Banana Pseudostem Hydrolysate

The sugars in the BPS hydrolysate samples were analysed using HPLC on a Shimadzu Prominence 20 HPLC system (Shimadzu, Kyoto, Japan) equipped with a Rezex RHM-monosaccharide H+ column (300 × 7.8 mm) fitted with a 5 micron Rezex organic guard column (Phenomenex, USA). Detection of eluents was done by using a refractive index detector, RID 10A (Shimadzu, Kyoto, Japan). The column temperature was maintained at 85°C, and analytes were eluted using deionised water at a flow rate of 0.6 ml/minute. Peak detection and integration were done using LC Solution software from Shimadzu (Kyoto, Japan), and peak heights were used for calculations of sugar concentrations. Known concentrations of glucose, xylose, and cellobiose were used as standards to determine the concentration of individual sugars in BPS hydrolysates.

The calculation of the glucose yield or saccharification yield as a percentage of cellulose converted into glucose (% digestibility) during enzymatic hydrolysis was done according to the method by Matsakas et al. [27].

(4) $$\begin{array}{c} \eta =\;100*\left(\frac{C_{\text{glucose }}*V_{\text{liquid}}*0.90}{m_{\text{solids }}*x_{\text{cellulose}}}\right), \end{array}$$

where *C*_glucose_ is the glucose concentration minus any glucose present at the beginning of hydrolysis (g/L) as determined by HPLC, *V*_liquid_ is the volume of liquid (*L*) used in saccharification, 0.90 is the correction factor for the conversion of cellulose to glucose, *x*_cellulose_ is the mass fraction of cellulose (expressed in dry biomass, g/g), and *m*_solids_ is the mass of dry solids (g/L).

### 2.8. Selected Saccharification Condition and Cultivation of Yeast

The fermentation medium used was hydrolysate from 15% loading of alkaline pretreated BPS hydrolysed with an increased dosage of crude cellulases (66.7 mg protein/g d.s equivalent to 20 FPU/g d.s) from *T*. *longibrachiatum* LMLSAUL 14-1 to achieve high sugar concentration. After 48 h of saccharification, hydrolysate of alkaline pretreated BPS was obtained by centrifuging the slurry at 3834 ×g with temperature maintained at 4°C for 30 minutes (Beckman Coulter Allegra X-22R refrigerated benchtop centrifuge). The glucose content of the hydrolysate was determined to be 74 g/L using HPLC. Bioethanol was produced through separate hydrolysis and fermentation (SHF). For inoculum preparation, *Saccharomyces cerevisiae* UL01 isolated from fermented sorghum was inoculated into 50 ml of the YPD (1% yeast extract, 2% peptone, and 2% dextrose) medium contained in 100 mL Erlenmeyer flasks and incubated at 30°C with shaking at 150 rpm for 14 hours (Incubator Shaker Series, New Brunswick, Excella E25R).

#### 2.8.1. Separate Hydrolysis and Fermentation

A 100 mL fraction of the fermentable sugars obtained from both acid pretreated BPS prehydrolysate and enzymatic alkaline pretreated BPS hydrolysate in 250 mL Erlenmeyer flasks was supplemented with 0.2 g K_2_HPO_4_, 0.7 g KH_2_PO_4_, 0.1 g NH_4_SO_4_, and 0.15 g yeast extract following a modification of the procedure given by Thakur et al. [37]. pH of the BPS hydrolysate medium was adjusted to 5.0, and it was autoclaved at 121°C and 15 psi for 15 minutes (HL-340 Vertical Type Steam Sterilizer, Taiwan) prior to inoculation. The BPS hydrolysate medium was allowed to cool and thereafter inoculated with *S*. *cerevisiae* UL01 to initial OD_600nm_ of 0.4 (*t*_0_) and incubated at 30°C and 100 rpm for 48 hours. The progress of fermentation was monitored through periodic sampling, and the samples were filtered through nonsterile 0.22 *μ*m syringe filter membranes prior to glucose and ethanol analysis.

#### 2.8.2. Ethanol Analysis in Fermented Hydrolysate

Ethanol was analysed by capillary gas chromatography using Shimadzu GC-2010 Plus equipped with autoinjector AOC 20i (Shimadzu) and an AOC 20S (Shimadzu) autosampler with a flame ionization detector (FID) and Zebron ZB-WAXplus 30 M (Phenomenex, USA) column (30 m, 0.25 mm ID and film thickness of 0.25 *μ*m). Nitrogen was used as the carrier gas at a flow rate of 17.6 mL/minute. The oven temperature was initially maintained at 40°C for 1 minute, then increased to 140°C at a rate of 20°C/min, and further increased to 200°C at a rate of 50°C/min and maintained at this temperature for 3 minutes. The injection temperature was 200°C, and the injection volume was 1 *μ*L. A split injection mode with a split ratio of 10 was applied. Ethanol was detected using a FID at 250°C. Absolute ethanol was used for the preparation of standard concentrations (v/v). Peaks' detection and integration were done using GC Solution software from Shimadzu (Kyoto, Japan). Peak heights were used to determine the unknown concentrations of ethanol in fermented BPS hydrolysate.

The conversion of cellulose to ethanol (%) was calculated based on the weight of biomass using the method given by Lu et al. [38]:

(5) $$\begin{array}{c} \text{ethanol yield }\left(\text{\%}\right)=\frac{\left[\text{EtOH}\right]}{\left(f\mathrm{*biomass*}1.111*0.51\right)}\times \;100, \end{array}$$

where [EtOH] is ethanol at the end of the fermentation minus any ethanol available in the medium (g/L) at time 0; *f* is the cellulose fraction of dry BPS biomass (g/g); biomass is the dry biomass concentration at the beginning of the fermentation (g/L); 0.51 is the conversion factor for glucose to ethanol based on stoichiometric biochemistry of yeast; and 1.111 is the conversion factor of cellulose to equivalent glucose.

### 2.9. Statistical Analysis

All experiments were performed in triplicate. The data generated were statistically analysed by a two-way analysis of variance (ANOVA) test for saccharification experiments using MS Excel 2010. Differences were considered significant when probability value *p* was <0.05. Error bars in the graphs represent standard deviation (SD).

## 3. Results

### 3.1. Pretreatment and Chemical Compositional Changes of the Banana Pseudostem

Banana pseudostem is a clustered cylindrical aggregation of leaf stalk bases. BPS contains polymers, such as cellulose, hemicellulose, and lignin [39]. The ultimate goal for applying different pretreatments of BPS was to determine and select the pretreatment that modifies the structural and chemical characteristics of the biomass to enhance its susceptibility to enzymatic hydrolysis. Pretreatment introduced changes in the structure of the BPS and opened the structure to expose the carbohydrate polymers by allowing improved enzyme access to the biomass, which leads to improved enzymatic hydrolysis (saccharification). The amounts of the three polymers found in the untreated and pretreated BPS are shown in Table 1. The pretreatment results showed an increase in the percentage of cellulose (Table 1) and a loss of hemicellulose as well as lignin (Tables 1 and 2). The loss of lignin is desirable since it has been shown that lignin binds and limits the accessibility of cellulases to cellulose [40].

Table 2 shows the effect of different pretreatments on the solubilisation of the BPS and the amount of sugars (i.e., glucose) been released in the process.

Both thermo-alkaline and thermo-acid pretreatments solubilised 60% of hemicellulose present and removed about 50% of lignin (Table 2). Alkaline and acid pretreatments improved cellulose availability by 114% and 97%, respectively. Hot water pretreatment of the BPS resulted in 41% hemicellulose solubilisation, 42% lignin removal, and very little change in the cellulose content.

### 3.2. Characterization of the Untreated and Pretreated BPS

FTIR spectroscopy was used to study the chemical, structural, and conformational variations introduced by the pretreatment of BPS. Lignocellulosic biomass contains many O-H bonds due to the presence of cellulose, hemicellulose, and lignin. During FTIR analysis, the covalent bonds of the functional groups absorb a certain amount of energy from infrared radiation, causing the bonds to stretch. The stretching of the bonds leads to an increase in peak intensity indicated by either sharpness or broadness [41].

**Figure 2:**
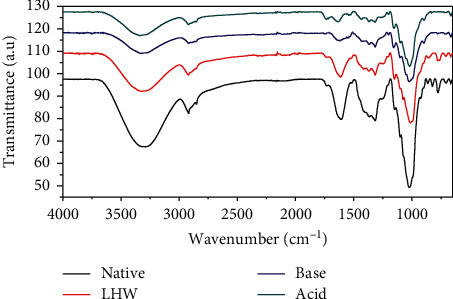
FTIR spectra of the banana pseudostem. 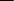, native (untreated); 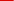, LHW (liquid hot water); 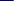, base (3% NaOH); 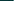, acid (5% H_2_SO_4_).

The FTIR spectra of the native (untreated) and pretreated BPS are shown in Figure 2. The spectrum of the native BPS shows distinct peaks/bands at various wavenumbers with strong broad bands occurring at 3363 cm^−1^, 2931 cm^−1^, 2794 cm^−1^, 1619 cm^−1^, 1263 cm^−1^, and 1010 cm^−1^ and multiple small peaks at 863 cm^−1^, 759 cm^−1^, 725 cm^−1^, and 718 cm^−1^. The spectra of all pretreated BPS fibres are similar to those of the native BPS and exhibit common peaks and values, which indicate the similarity between samples. However, chemical modification through pretreatment has decreased the peaks' intensities, and some peaks completely disappeared. For instance, the peak at wavenumber 2794 cm^−1^ disappeared in all the pretreated BPS samples, suggesting solubilisation of hemicellulose (Table 2). There was a decrease in peak intensities of the pretreated BPS samples at wavenumbers 3363 cm^−1^, 2931cm^−1^, 1619 cm^−1^, 1235 cm^−1^, and 1010 cm^−1^ indicating fewer functional groups such as O-H, C-H, and C=C that are associated with stretching vibrations mainly in cellulose and hemicellulose or lignin.

### 3.3. Hydrolysis of the Banana Pseudostem

High solid loading of BPS during enzymatic hydrolysis was selected to evaluate the efficiency of the crude cellulases from the *Trichoderma* species at moderate dosage and to establish the “high-solid effect” defined as a decrease in cellulose conversion yield or efficiency as solid loading increases [42]. All the pretreated BPS were exposed to enzyme digestibility assessment (saccharification) using crude cellulases from *T*. *harzianum* LMLBP07 13-5 and *T*. *longibrachiatum* LMLSAUL 14-1 at a concentration of 10 FPU/g d.s (equivalent to 29.3 mg protein/g d.s).

**Figure 3:**
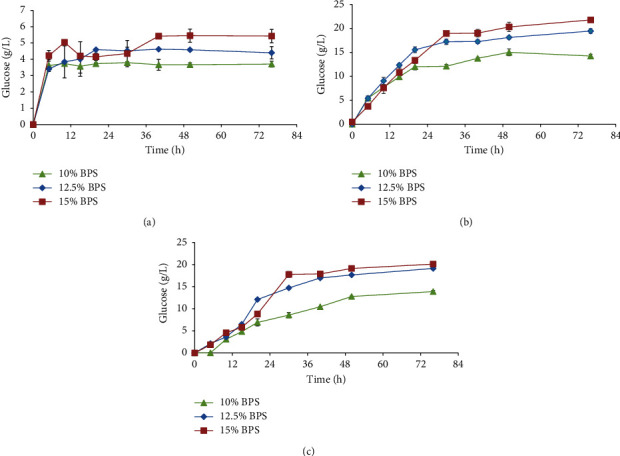
Time course of the pretreated BPS by the crude enzyme from *T. harzianum* LMLBP07 13-5: (a) acid; (b) LHW; (c) alkaline.

Acid pretreated BPS exhibited recalcitrance towards the enzymatic hydrolysis by cellulases from *T*. *harzianum* LMLBP07 13-5. When compared with the LHW and alkaline pretreatment of BPS, there was poor release of glucose at all solid/substrate loadings. The concentration of glucose ranged from 3.8 to 5.4 g/L with higher concentration attained at 15% substrate loading (Figure 3(a)). When the LHW pretreated BPS was exposed to enzymatic hydrolysis, released glucose ranged from 14.2 to 22 g/L after 76 h (Figure 3(b)). The hydrolysis rate for the LHW pretreated BPS was faster than for both chemically pretreated BPS. At 20 h, the amount of glucose released from 12.5% substrate loading was 16 g/L higher than the alkaline pretreated BPS with the same substrate loading. The rate of hydrolysis slowed as substrate loading increased to 15%. The hydrolysis of the alkaline pretreated BPS released glucose in the range of 13.9 to 20.1 g/L (Figure 3(c)). In all treatments, the highest concentration of glucose was attained at 15% solid loading of BPS. The concentration of glucose increased proportionally with an increase in substrate loading. At 76 h, the hydrolysis of both LHW and alkaline pretreated BPS at all substrate loadings (10–15%) showed no significant differences with the amount of glucose released.

The cellulose conversion to glucose of the BPS varied between the treatments. As shown in Table 3, LHW pretreated BPS had higher cellulose conversion to glucose, i.e., higher enzyme digestibility ranging from 50 to 51%. Alkaline pretreated BPS was the second most digestible material with cellulose conversion between 24 and 23%. Both LHW and alkaline pretreated BPS exhibited an increase in cellulose conversion to glucose up to a solid loading of 12.5%. Further solid loading of 15% BPS decreased the conversion of cellulose to glucose. There was poor cellulose conversion to glucose in the acid pretreated BPS which exhibited digestibility of 7% in all solid loadings.

**Figure 4:**
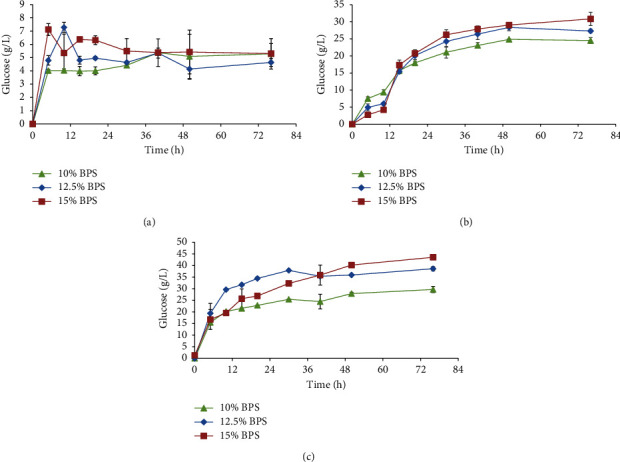
Time course of the pretreated BPS by the crude enzyme from *T. longibrachiatum* LMLSAUL 14-1: (a) acid; (b) LHW; (c) alkaline.

The hydrolysis of the acid pretreated BPS by cellulases from *T. longibrachiatum* LMLSAUL 14-1 is shown in Figure 4(a). Most of the sugar was released within the first 5 to 10 hours of incubation, and a further increase in hydrolysis time did not improve the glucose concentration significantly. The hydrolysis of LWH by crude cellulases from *T*. *longibrachiatum* LMLSAUL 14-1 showed a lag phase up to 10 h, a period in which the hydrolysis rate of the enzymes slowed. After 10 h, the hydrolysis rate increased, thereby releasing glucose that ranged from 25 to 31 g/L at 76 h (Figure 4(b)). The cellulases produced by *T*. *longibrachiatum* LMLSAUL 14-1 were more effective in hydrolysing alkaline pretreated BPS (Figure 4(c)). The hydrolysis rate for the alkaline pretreated BPS was faster than the rate for the LHW pretreated BPS. The enzymatic hydrolysis released glucose in the range of 29.7 to 43.5 g/L. The amounts of glucose released from the hydrolysis of the alkaline pretreated BPS were significantly higher than the other pretreated BPS. Glucose obtained from LHW and alkaline pretreatments was directly proportional to the solid loading of the BPS.

A significant improvement in the enzymatic conversion of the LHW pretreated BPS was evident when compared with other pretreatments (Table 4). The highest glucose yields or percent of saccharification ranged from 87 to 73%, which was attained by using 10 to 15% LHW pretreated BPS solid loading that was hydrolysed by crude cellulases from *T. longibrachiatum* LMLSAUL 14-1. In comparison to the digestibility of the LHW BPS that remained high, there was a parallel proportional decrease in alkaline BPS conversion to glucose with increasing solid loading up to 15%. Similarly, in the alkaline pretreated PBS, the glucan conversion efficiency decreased only after 12.5% solid loading (Table 4). Although the cellulose or glucan conversion efficiency of the alkaline pretreated PBS was lower than the LHW pretreated BPS, the hydrolysis of the alkaline pretreated BPS released more sugar than all other pretreated PBS at the end of the reaction. Acid pretreated BPS cellulose conversion to glucose remained poor in all solid loadings, and therefore, lower glucose concentration was obtained.

Based on the glucose concentration obtained from the hydrolysis reactions of the alkaline pretreated BPS with *T*. *longibrachiatum* LMLSAUL 14-1 cellulases, this reaction was chosen for further use in fermentation studies.

### 3.4. Separate Hydrolysis and Fermentation of Enzymatic Alkaline BPS Hydrolysate

**Figure 5:**
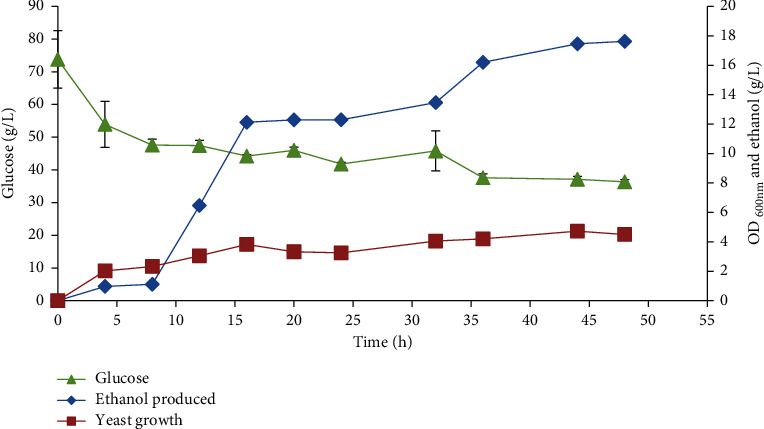
Time course of the separate hydrolysis and fermentation of the alkaline pretreated BPS saccharified with *T. longibrachiatum* LMLSAUL 14-1 cellulases.

Acid hydrolysis of lignocellulosic biomass has been reported to yield high concentrations of fermentable sugars, but it produces toxic substances that requires the neutralization step prior to fermentation. This adds more cost to bioethanol production [43]. As opposed to acid hydrolysis, enzymatic hydrolysis uses very mild reaction conditions and produces very little toxic compounds [44]. After hydrolysis of the alkaline pretreated BPS with *T*. *longibrachiatum* LMLSAUL 14-1 cellulases, the amount of glucose released reached 74 g/L. The fermented alkaline pretreated BPS enzymatic hydrolysate was shown containing 17.6 g/L concentration of ethanol at the end of fermentation by converting 51% of available glucose, with residual glucose of 36.3 g/L (Figure 5). The fermentation was sluggish and incomplete. The ethanol yield was 60% of the theoretical maximum.

## 4. Discussion

### 4.1. Chemical Composition of the Banana Pseudostem

Different BPS portions, outer sheath, middle portion, and core portion (i.e., pith), were mixed and analysed for its polymer content. The cellulose content of the untreated BPS (24.5%, Table 1) was comparable to 20.1% reported by Guerrero et al. [45]. Several authors reported between 30 and 44% cellulose, 15 and 30% hemicellulose, and 6 and 12% lignin in the untreated BPS [13, 21, 37, 46–48]. The discrepancies in chemical composition may be attributed to the cultivar of the banana plant and climatic (geographic) conditions. Pretreatment of the BPS changed its chemical, physical, and morphological structure. This observation was evident as shown in by FTIR analysis (Table 1 and Figure 2). The results showed an increase in cellulose and partial loss of hemicellulose and lignin, particularly in chemically pretreated BPS compared to the hydrothermally (liquid hot water) pretreated BPS. The effect of acid and alkaline pretreatments on the reduction of hemicellulose and lignin in the BPS is consistent with the findings of de Souza et al. [48].

Available cellulose in the pretreated BPS with alkaline solution (3% NaOH) reported in this study was lower as shown in Table 1 when compared to 73.74% BPS cellulose obtained when higher alkaline concentration (4% NaOH) was used by Low et al. [36]. Hemicellulose at 8.35% and lignin at 10.1% are in agreement with our results. These authors also observed that prolonged soaking times and increased concentration of NaOH did not result in complete removal of lignin. The loss of hemicellulose in NaOH pretreated biomass is thought to be the result of a peeling mechanism, which removes the terminal sugar molecules one at a time from the reducing end [49]. The modification of biomass by pretreatment removes hydrogen bonding in the biomass network structure. The effects of pretreatment on the BPS chemical composition were found similar to those observed in agricultural residues. For example, pretreatment of wheat straw (WS) by hydrothermal and steam explosion increased the cellulose content and reduced the hemicellulose content [50, 51]. Zhang et al. [52] reported about 58% cellulose, 28.85% hemicellulose, and 17.75% lignin, which amounted to a 19.53% increase of cellulose and 13.98% decrease of lignin content with a small increase of hemicellulose after mild alkaline pretreatment (1% NaOH) of wheat straw. The higher cellulose content and reduced hemicellulose and lignin contents enhanced enzymatic saccharification. Motaung and Anandjiwala [28] also observed an increase of cellulose with the decrease of hemicellulose and lignin contents in chemically pretreated sugarcane bagasse. In addition, the authors also mentioned higher cellulose and hemicellulose contents in acid pretreated sugarcane bagasse (SB) than in alkaline pretreated SB.

### 4.2. Characterization of the Untreated and Pretreated Banana Pseudostem

FTIR spectroscopy was used to analyse the chemical, structural, and conformational variations introduced by pretreatment in the BPS. The broad band (peak) occurring at wavenumber 3363 cm^−1^ in Figure 2 was associated with the intermolecular O-H stretching vibrations of cellulose and 2931 cm^−1^ with C-H stretching absorption/vibration from –CH_2_ groups of cellulose and hemicellulose [53–55]. A peak at 1619 cm^−1^ is associated with C=C stretching of benzene rings in lignin and 1010 cm^−1^ with C-O-C stretching absorption between raw banana fibres [53]. According to Faix et al. [56], lignin compounds are characterized by the frequencies of guaiacyl units, corresponding to wavenumber 1269 cm^−1^ and G-ring and C=O stretched at 1140 cm^−1^. Both untreated and LHW pretreated BPS showed smaller peaks at 863 cm^−1^ and 759 cm^−1^. The peak between 800 cm^−1^ and 880 cm^−1^ is associated with CH in-plane deformation [57], while the peak at wavenumber 750 cm^−1^ signifies ArC-H out-of-plane deformation of lignin. The peaks at wavenumbers 687–625 cm^−1^ are associated with the out-of-plane bending vibrations of intermolecular H-bonded O-H groups and out-of-plane O-H bending [58].

In another study, FTIR analysis also revealed that dilute acid pretreatment of wheat straw alone was not sufficient for complete lignin removal, although complete removal of soluble lignin was attained as shown by a slight appearance or disappearance of a peak associated with both lignin types [59]. Barreto et al. [60] found that the vibration modes of functional groups of the pretreated banana fibre did not show differences compared with the main bands of the untreated banana fibre after alkaline treatment at concentrations ranging from 0.25 to 1% NaOH at 60–70°C for 6 hours. However, there were clear differences between the pretreated and untreated BPS biomass. The difference between the observation reported by Barreto et al. [60] and the finding in this study could be attributed to pretreatment conditions as the current study used a higher percentage of NaOH (3% w/v) and temperature (121°C) to BPS.

### 4.3. Hydrolysis of the Banana Pseudostem

The sole purpose of hydrolysis is to release sugars, mainly glucose (and xylose depending on the enzyme composition), which will be fermented in the subsequent steps. Enzymatic hydrolysis (saccharification) of lignocellulosic biomass is dependent on both the availability of cellulose or hemicellulose and the hydrolytic activity of the enzymes used. In this study, saccharification of BPS was carried out with an enzyme dosage of 10 FPU/g substrate (equivalent to 29.3 mg protein/g d.s). Substrate loading ranged from 10 to 15%. Based on reported findings by Lu et al. [61], Sun and Cheng [62], and Gregg and Saddler [35], cellulase dosage for effective hydrolysis could vary between 7 and 33 FPU/g substrate, depending on the type of substrate. Guerrero et al. [45] achieved hydrolysis of banana waste with 15 FPU/g cellulase dosage with a high solid loading (i.e., higher than 15% solid loading).

During hydrolysis of the BPS, the concentration of glucose has increased proportionally with increasing solid loading of pretreated BPS biomass. However, the cellulose or glucan conversion efficiency (i.e., glucose yield) to glucose increased up to 12.5% solid loading, and the efficiency reduced with further increases of solid loadings (Tables 3 and 4). The enzymatic release of the sugars from the acid pretreated BPS was poor because of the indigestibility of cellulose fibres, which may contain many crystalline regions on the cellulose surface. This observation is in agreement with the findings by Abraham et al. [63], who showed that oxalic acid hydrolysis solubilised amorphous regions in cellulose leaving more crystalline regions in the cellulose fibres. An increase in oxalic acid concentration up to 10% increased the crystallinity of the BPS, jute stem, and pineapple leaf fibre. A concentration of oxalic acid higher than 20% degraded cellulose fibres [63]. Another possible reason may be that the enzyme concentration used for hydrolysis is not optimal for achieving high glucose at a solid loading higher than 10%. For instance, Shimizu et al. [64] reported the hydrolysis of 2% solid loading (i.e., 0.1 g in 5 mL reaction volume) of acid (5% v/v H_2_SO_4_) pretreated BPS by commercial enzymes (15 FPU/g cellulase, Celluclast 1.5 L, Novozyme, and 15 U/g cellobiase, *β*-glucosidase, and Novozyme 188), which provided sufficient enzymatic activity to achieve a glucose yield of 20%.

Unlike saccharification of the acid pretreated BPS, enzymatic saccharification of LHW and alkaline pretreated BPS, which contained 40% and 63.0% holocellulose, respectively, by crude cellulases from *T*. *harzianum* LMLSABP07 13-5 and *T*. *longibrachiatum* LMLSAUL 14-1 was shown susceptible to enzymatic hydrolysis. The concentration of glucose (g/L) released from both LWH and alkaline pretreated BPS showed no significant differences after the hydrolysis by the crude enzyme from *T*. *harzianum* LMLSABP07 13-5.

An exception was the hydrolysis of the LHW pretreated BPS by crude cellulases from *T*. *longibrachiatum* LMLSAUL 14-1, which showed reduction in glucan conversion efficiency at a biomass loading of more than 10% (Table 4). Moreover, the glucan conversion efficiency of the LHW pretreated BPS was found higher than the other saccharified pretreated BPS. Under optimized conditions, LHW could be a pretreatment of choice because it was environmentally friendly (i.e., less harmful to the environment), safe, and also economical compared to the chemical pretreatment. The reduction in cellulose efficiency at high solid loading was in agreement with the findings by Guerrero et al. [45], who observed that higher solid loadings led to a higher glucose content, but led to low cellulosic conversion efficiency. A recent study by Lu et al. [61] showed the extent to which a biomass can be hydrolysed at high solid loading and yet still achieve high conversion efficiency. The study found that, at high solid loading between 10 and 20% of corn stover, the glucose yield remained unchanged. However, further increase in solid loading to 30% led to significant reduction in glucose yield. The high conversion efficiency at 20% solid loading of corn stover was attributed to ball milling pretreatment, thereby reducing the crystallinity and the degree of polymerization of the polymers in corn stover [61]. The decrease in cellulosic conversion efficiency to glucose is highly undesirable as it diminishes the significant advantages of working at high solid loadings [65]. The enzyme-substrate interaction becomes ineffective due to the higher viscosity of the slurry. Ghose [66] found that high viscous slurry restricts enzyme movement and makes hydrolysis sites inaccessible.

Souza et al. [48] reported high crystallinity of the alkaline pretreated BPS when compared to the acid pretreated BPS, which led to poor enzymatic conversion of alkaline pretreated BPS cellulose to glucose. As a result, high reducing sugar concentrations (33.74 g/L) were obtained from the acid pretreated BPS compared to 19.4 g/L obtained from the alkaline pretreated BPS in the Souza et al.'s study [48]. The high digestibility of the acid pretreated BPS reported by Souza et al. [20, 48] could be due to the low acid concentration (i.e., 2% H_2_SO_4_) used as compared to 5% H_2_SO_4_ used in this study. As concentration of acid increases, the severity of the pretreatment also increases; this may cause solubilisation of hemicelluloses and more amorphous regions within cellulose, making it indigestible.

According to El-Zawawy et al. [24], the concentration of glucose in the hydrolysate was influenced by the type of pretreatment used and the type of hydrolysis. The authors utilised the steam explosion pretreatment of banana plant waste followed by the enzymatic hydrolysis of the plant waste by cellulases from *T*. *reesei* ATCC 26921, which released a higher concentration of glucose. Filho et al. [23] found that the alkaline pretreated BPS was susceptible to enzyme hydrolysis, which resulted in a 23-fold increase of fermentable sugars as compared to an 8-fold increase obtained through acid hydrolysis. Another study on the BPS, Thakur et al. [37], also found that alkaline (4% NaOH, slightly higher than the concentration used in the current study) pretreatment of BPS and WS yielded higher concentrations of reducing sugars compared to acid (5% H_2_SO_4_) and biological (fungal) pretreatments. Chidi et al. [67] also found that the alkaline (2% NaOH) pretreatment of BPS combined with microwave irradiation (170 W, 10 minutes) yielded the highest concentration of reducing sugar for the production of bioethanol.

Alrumman [68] investigated the effect of substrate loading of palm leaves at concentration 1–8% and found that a 4% substrate loading of alkaline pretreated date palm leaves produced the highest sugar concentration during saccharification. Xu et al. [69] inferred that alkaline pretreatment technology is a promising pretreatment method of lignocellulosic biomass resulting in relatively low lignin, leading to improved enzymatic digestibility. Technoeconomic and environmental studies conducted by Duque et al. [4] listed banana pseudostem amongst other agricultural residues, such as sugarcane bagasse, corn cob, and rice husk, as a potential feedstock for the production of bioethanol. It was reported that future work should focus on developing efficient enzyme cocktail formulations to hydrolyse different types of lignocellulosic biomass to make cellulosic ethanol a cost-effective process [45]. It is imperative to select a suitable pretreatment method that maximises the concentration of fermentable sugar after enzymatic saccharification in order to improve the efficiency of cellulosic bioethanol production [70]. Given the amount of polymers in the BPS, particularly cellulose and hemicellulose (Table 1) and solubilised sugars (Table 2), which are present in the liquid fraction after the pretreatment, necessitates further studies that combine sugars in the liquors and sugars from solid fraction for fermentation in order to achieve higher ethanol yields and make the process cost effective.

### 4.4. Production of Ethanol through Separate Hydrolysis and Fermentation

During saccharification, 74 g/L of glucose was produced in enzymatic alkaline BPS hydrolysate. The glucose levels reported in this study were higher than the levels obtained from 5 g pretreated banana pseudostem (15.3 g/L sugar) reported by Thakur et al. [37]. The production of ethanol is strongly dependent on the growth of the fermenting organism. *Saccharomyces cerevisiae* is known to consume glucose rapidly during growth and tolerate high levels of ethanol. However, poor growth of *S. cerevisiae* UL01 in the early stages of fermentation in enzyme hydrolysate (after alkaline pretreatment) led to high levels of residual sugar at the end of fermentation. It is desirable for a fast fermentation process of ethanol production since it results in improved ethanol productivity, which has a direct impact on the economics of the fermentation process and its commercial feasibility [71].

The sluggish and incomplete fermentation of alkaline pretreated BPS enzymatic hydrolysate yielded 18 g/L ethanol with a 60% conversion of cellulose to ethanol and productivity of 0.01 g/L/h. The incomplete fermentation could be attributed to residual inhibitory substances such as inorganic salts and phenolics that adsorbed onto the polymer fibres after the BPS solid washing step (i.e., ineffective washing) since no detoxification process was done. Thakur et al. [37] also produced ethanol from enzymatic hydrolysates of different pretreated BPS by *S*. *cerevisiae* NCIM 3570. The authors found that ethanol produced in the enzymatic hydrolysate of the alkaline pretreated BPS reached a maximum of 3.8 g/L with an ethanol yield of 0.35 g/g, which were higher than the levels obtained from the acid pretreated BPS (1.9 g/L and 0.20 g/g). Although enzymatic hydrolysate of the fungal pretreated BPS yielded a low ethanol concentration (2.0 g/L), the ethanol yield was higher (0.40 g/g) than the chemically pretreated BPS [37]. Kusmiyati and Sukmaningtyas [72] reported 4.32 g/L ethanol produced from the alkaline (8% NaOH) pretreated BPS at a 10% solid loading in simultaneous saccharification and fermentation (SSF), while El-Zawawy et al. [24] reported low ethanol concentrations (<3.5 g/L) from banana plant waste.

## 5. Conclusions

Alkaline pretreatment of BPS resulted in a high holocellulose content. Enzymatic hydrolysis of an alkaline pretreated BPS found releasing more glucose concentration, and this was shown to be followed by the LHW pretreated BPS, irrespective of the source of cellulases used. A LHW pretreated BPS exhibited the highest saccharification efficiency (glucan conversion efficiency) that was followed by the alkaline pretreated BPS, irrespective of cellulases used. However, high cellulose (glucan) conversion efficiency was achieved with crude cellulases from both fungal species, when the LHW pretreated BPS was hydrolysed. Although the LHW pretreatment of BPS was shown to be a promising method that can potentially substitute the chemical methods, it requires further optimization. The utilization of the BPS for cellulase and bioethanol production has the potential to lower the cost of cellulosic ethanol. The incomplete fermentation observed here requires further optimization and the use of a more robust yeast strain with improved fermentative properties.

## Figures and Tables

**Table 1:** Chemical composition of the untreated and pretreated banana pseudostem.

BPS and pretreatment	Cellulose (%)	Hemicellulose (%)	Lignin (%)
Untreated	24.47 ± 0.84	22.56 ± 1.66	14.14 ± 1.59
3% NaOH	52.32 ± 2.88	10.84 ± 1.59	8.68 ± 0.46
5% H_2_SO_4_	48.17 ± 0.35	9.88 ± 1.64	8.31 ± 1.69
Hot water	25.44 ± 0.31	15.02 ± 1.19	9.25 ± 0.07

NB: standard deviation (SD) ± values of three independent repeats.

**Table 2:** Effect of pretreatment methods on solubilisation components of the banana pseudostem.

Banana pseudostem pretreatments (1 : 10 of mass to liquid volume)	Mass before pretreatment (*W*_untreat_, g/L)	Mass after pretreatment (*W*_pretreat_, g/L)	Prehydrolysate (glucose, g/L)	Percentage of solubilised components of the banana pseudostem (%), equation (1)	
				Hemicellulose	Lignin
Alkaline	150	124.5	17	60.1	51.0
Acidic	150	118.5	21	65.4	54.0
Liquid hot water	150	132.0	12	41.4	42.4

**Table 3:** Summary of the enzymatic hydrolysis of pretreated banana pseudostems at varying solid loadings by crude enzymes produced by *Trichoderma harzianum* LMLBP07 13-5.

Pretreatment	BPS biomass loading (% w/v)	Crude cellulase loading (FPU/g d.s)	Glucose produced (g/L)	Time (h)	Cellulose conversion to glucose (% w/w), equation (4)
5% (v/v) H_2_SO_4_	10	10	3.8 (±0.06)	30	7.1 (±0.11)
	12.5	10	4.6 (±0.02)	40	6.9 (±0.03
	15	10	5.4 (±0.40)	40	6.8 (±0.50)

LHW	10	10	14.2 (±0.36)	76	50.2 (±0.59)
	12.5	10	19.5 (±0.49)	76	55.2 (±0.71)
	15	10	21.8 (±0.50)	76	51.4 (±0.79)

3% (w/v) NaOH	10	10	13.9 (±0.31)	76	24.1 (±0.54)
	12.5	10	19.1 (±0.21)	76	26.3 (±0.30)
	15	10	20.1 (±0.16)	76	23.1 (±0.19)

Standard deviation ± values of three independent repeats.

**Table 4:** Summary of enzymatic saccharification of pretreated banana pseudostems at varying solid loadings by crude enzymes produced by *Trichoderma longibrachiatum* LMLSAUL 14-1.

Pretreatment	BPS biomass loading (% w/v)	Crude cellulase loading (FPU/g d.s)	Glucose produced (g/L)	Time (h)	Cellulose conversion to glucose (% w/w), equation (4)
5% (v/v) H_2_SO_4_	10.0	10	5.4 (±1.04)	40	10.1 (±1.95)
	12.5	10	7.4 (±0.36)	10	11.1 (±0.57)
	15.0	10	7.1 (±0.45)	5	8.9 (±0.56)

LHW	10.0	10	24.5 (±0.84)	76	86.7 (±1.25)
	12.5	10	27.3 (±0.53)	76	77.3 (±0.86)
	15.0	10	30.8 (±1.93)	76	72.6 (±1.83)

3% (w/v) NaOH	10.0	10	29.7 (±1.31)	76	51.1 (±2.26)
	12.5	10	38.6 (±0.85)	76	53.1 (±1.17)
	15.0	10	43.5 (±0.70)	76	50.0 (±0.81)

Standard deviation ± values of three independent repeats.

## Data Availability

The experimental data used to support the findings of this study are available from the corresponding author upon request.
